# Anti-hyperuricemic and Anti-inflammatory Effects of *Marantodes pumilum* as Potential Treatment for Gout

**DOI:** 10.3389/fphar.2020.00289

**Published:** 2020-03-17

**Authors:** Eldiza Puji Rahmi, Endang Kumolosasi, Juriyati Jalil, Khairana Husain, Fhataheya Buang, Amirul Faiz Abd. Razak, Jamia Azdina Jamal

**Affiliations:** ^1^Drug and Herbal Research Centre, Faculty of Pharmacy, Universiti Kebangsaan Malaysia, Kuala Lumpur, Malaysia; ^2^Faculty of Medicine, Universitas Pembangunan Nasional “Veteran”, Jakarta, Indonesia

**Keywords:** *Marantodes pumilum*, *Labisia pumila*, hyperuricemia, monosodium urate crystals, xanthine oxidase inhibitor, pro-inflammatory cytokines, prostaglandin E_2_

## Abstract

*Marantodes pumilum* (Primulaceae) has been used in Malaysian folk medicine to help women regain strength after delivery and for “sickness in the bones.” It was previously revealed that its extracts inhibited xanthine oxidase (XO) activity *in vitro*. The leaves and roots of *M. pumilum* var. *alata* (MPA), var. *pumila* (MPP), and var. *lanceolata* (MPL) were individually extracted in ethanol (80%). The anti-hyperuricemic activity was initially assessed by XO inhibition with a spectrophotometric *in vitro* assay. The most active extract was further investigated on hyperuricemic rat model induced by potassium oxonate to determine serum uric acid levels and liver XO effect. The *in vitro* anti-inflammatory activity was carried out on monosodium urate (MSU) crystal-induced pro-inflammatory cytokines (i.e., interleukin (IL)1α, IL-1β, IL-6, IL-8, and tumor necrosis factor (TNF)-α) secretion using human peripheral blood mononuclear cells and ELISA technique, and prostaglandin E_2_ (PGE_2_)secretion using radioimmunoassay. The active extract was then investigated on gout-induced inflammation with MSU crystals to determine pro-inflammatory cytokines and PGE_2_ secretion levels in the synovial fluid of rat knee joint. Quantitative analysis using validated HPLC was performed on the extracts to determine presence of bioactive flavonoids. The findings revealed that extract of MPP leaves gave the highest inhibitory activity on XO (IC_50_ 130.5 μg/mL) compared to other extracts tested. However, all extracts possessed significantly lower activity compared to allopurinol (IC_50_ 0.13 μg/mL). Oral administration of MPP leaf extract (200 mg/kg) significantly reduced serum uric acid level in hyperuricemic rats in time-dependent manner to the baseline level and it was as effective as allopurinol (5 mg/kg). The extract also inhibited liver XO activity (25%) compared to allopurinol (45%). *In vitro* anti-inflammatory assay showed that extract of MPP roots inhibited MSU crystals-induced secretion of IL-1α, IL-1β, IL-8, TNF-α, and PGE_2_ with IC_50_ values of 36, 25, 38, 18, and 46 μg/mL, respectively. Oral administration of the MPP root extract (200 mg/kg) significantly decreased IL-1α, IL-1β, IL-6, TNF-α, and PGE_2_ levels in rat’s synovial fluid as effective as indomethacin. There were no significant body weight changes of all experimental animals. MPP extracts showed presence of myricetin, quercetin and kaempferol. Myricetin was detected with values of 0.2 and 0.6 mg/g for root and leaf extracts, respectively. The anti-hyperuricemic of MPP leaf and anti-inflammatory of MPP root indicated that MPP may be promising for complementary therapy of gout.

## Introduction

Gout is the most common inflammatory arthritis with an increasing incidence and prevalence worldwide (Kuo et al., 2015). According to the Global Burden Disease 2010 study, the burden of gout has significantly increased by 49% and has risen by the increasing of the aging populations (Smith et al., 2014). Gout occurs predominantly among men and older women population. Generally, the incidence is 2–6 times higher in men rather than in women with the increase of age (Kuo et al., 2015). Oceanian countries, especially ethnic groups of Taiwanese aboriginals and Maori were reported to have the highest prevalence of gout with the percentage of 2–20 and 13.9%, respectively (Tu et al., 2015). In Malaysia, male and female ratio of gout was 8:1 and the most likely ethnic group to develop gout in Malaysia was Malay (72%), followed by Indian (20%) and Chinese (8%) (Mohd et al., 2011). Gout was also reported to be associated with a number of significant co-morbidities, including cardiovascular disease, chronic kidney disease, obesity and type 2 diabetes (Robinson and Horsburgh, 2014). Moreover, recent studies reported that gout was known as an independent risk factor of cardiovascular disease. Current studies by Fernandez and Markenson (2015) and Clarson et al. (2015) reported that there was a significant increase in cardiovascular disease and mortality in patient with gout and hyperuricemia. Thus, it is important to optimize the management of gout in order to minimize the disease burden and also to reduce the development of co-morbidities.

Gout occurs due to the deposition of uric acid crystals in tissues, tendons and joints which causes inflammatory response (Chen et al., 2011). Therefore, the treatment approaches of gout are mainly mechanism-based therapies that reduce uric acid levels and inflammation, such as anti-hyperuricemic and anti-inflammatory agents (Dalbeth and Stamp, 2007). However, the use of these drugs has been associated with several adverse effects that may affect patients’ compliance (de Souza et al., 2012). Besides the allopathic medicine, herbal therapy and phytopharmaceuticals are another effective alternative medication. Therefore, the search for alternative treatment of gout has prompted researchers to investigate the beneficial value of natural products.

Natural products, including minerals, plants, animals, microorganisms, and marine organisms, have been used as drugs since prehistoric times. There are so many plants used for traditional medicine, such as in Ayurvedic Medicine, Chinese Traditional Medicine, Unani Medicine, Traditional Korean Medicine, Kampo, etc., which probably have been used for hundreds or thousands of years for therapeutic purposes and need to be proven for its efficacy and safety to fulfill the modern standards of therapeutic agents (Chin et al., 2006; Subramoniam et al., 2013). Considering their incomparable chemical diversity and novel mechanisms of action, natural products have continued to play a pivotal role in any drug development and research programs. Thus, the research in natural products continues to explore potential therapeutic activities as well as variety of active components, which may lead to development of new drugs by pharmaceutical industry (Lahlou, 2013).

Along this way, several plant extracts and their isolated constituents have been reported for their anti-gout activities including anti-hyperuricemic and anti-inflammatory *in vitro* and *in vivo*. The most famous example of natural product as treatment for gout is colchicine, an alkaloid from plant *Colchicum autumnale*. It has been used clinically for treatment of gouty inflammation (Ahern et al., 1987). Recent study by Mamat et al. (2014) investigated a total of 129 extracts of different plant parts of twelve Primulaceae species for XO inhibitory activity *in vitro*. Among all extracts tested, 14 extracts were found to possess more than 50% inhibition. The results showed that the dichloromethane extract of MPA roots gave the strongest activity with IC_50_ value of 4.8 μg/mL as compared to other extracts tested. Previous scientific studies also revealed various biological activities of *M. pumilum* including anti-oxidant and anti-inflammatory activities (Karimi et al., 2013).

*Marantodes pumilum* (Blume) Kuntze (Primulaceae) is previously known as *Labisia pumila* (Blume) Fern.-Vill under the family of Myrsinaceae (The Plant List, 2013). *M. pumilum* is locally known in Malaysia as kacip fatimah, akar fatimah, selusuh fatimah, rumput siti fatimah, pokok pinggang, belangkas hutan, rumput palis, tadah matahari, mata pelanduk rimba, and sangkoh (Burkill, 1966; Sunarno, 2005; Abdullah et al., 2013, Supplementary Table S1). It has been used in traditional medicine especially among Malay women as a decoction for reproductive-related conditions, including to induce and ease childbirth, and help women to tighten the birth canal, strengthen the abdominal muscle, as well as regain strength after delivery. It is also used to improve the menstrual irregularities and alleviate menstrual cramps. The preparations have also been used for flatulence, dysentery, gonorrhoa and “sickness in the bones.” The regular consumption of this decoction is believed to help maintain and improve health and wellness (Burkill and Haniff, 1930; Burkill, 1966; Bodeker, 2009).

Several phytochemical studies have been done to identify and isolate the chemical constituents from *M. pumilum*. Chua et al. (2011) reported nine flavonol derivatives of quercetin, myricetin and kaempferol, and two flavanol derivatives of cathecin and epigallocatechin which were identified from 40% of methanol (MeOH) fraction of MPA leaves. Resorcinol derivatives in *M. pumilum* have been reported by previous studies, such as (*Z*)-5-(pentadec-4′-enyl)-resorcinol, (*Z*)-5-(pentadec-8′-enyl)-resorcinol and (*Z*)-5-(pentadec-10′-enyl)-resorcinol were isolated from MPA roots (Jamia et al., 1998). Strong anti-oxidative compounds such as β-carotene and ascorbic acid were also found in *M. pumilum* (Chua et al., 2011; Karimi et al., 2011a). Recent studies by Hairi et al. (2018) reported demethylbelamcandaquinone B isolated from aqueous extract of MPA leaves.

The popularity of *M. pumilum* as traditional medicine is undeniable, particularly in Malaysia. The fact that it is used to treat “bone sickness” and was reported to exhibit anti-inflammatory and xanthine oxidase inhibitory activities initiated the idea that *M. pumilum* might be potential as antigout. However, to the best of our knowledge, there is still lack of studies, particularly *in vivo* studies, have been conducted to investigate whether *M. pumilum* possesses antigout activity in terms of its ability to reduce uric acid levels and treat crystal-induced inflammation that is related to gouty condition. Realizing the facts that *M. pumilum* showed a potential antigout effects, therefore, this study is prompted to further investigate the anti-hyperuricemic and anti-inflammatory effects of *M. pumilum in vitro* and *in vivo.*

## Materials and Methods

### Chemical and Reagents

HPLC-grade MeOH and acetonitrile were obtained from Merck (Darmstadt, Hesse, Germany), HPLC-grade orthophosphoric acid was obtained from Fisher Scientific (Loughborough, Leicestershire, United Kingdom) while pure water for HPLC was obtained from ultrapure water system machine (PureLab, United States). Dexamethasone phosphate was obtained from Duopharma (M) Sdn. Bhd., Malaysia. Lymphoprep was obtained from Axis-Shield PoC AS (Oslo, Norway), while indomethacin, allopurinol, potassium oxonate, uric acid, xanthine substrate, unlabelled PGE_2_, anti-PGE_2_, HEPES, FBS, penicillin streptomycin solution and RPMI-1640 medium containing L-glutamine were obtained from Sigma Chemical Co. (St. Louis, MO, United States). Xanthine oxidase from bovine milk (20 U/mL) was purchased from Roche Diagnostic GmbH (Mannheim, Baden-Württemberg, Germany). IL-8 ELISA kits were purchased from Abnova, Germany. Kits for determination of xanthine oxidase activity and plasma uric were purchased from BioVision (Milpitas, CA, United States), while all of the other kits were purchased from Cayman, United States. Radiolabelled PGE_2_ ([^3^H]-PGE_2_, 50 μCi/mmol) and liquid scintillation cocktail were purchased from Perkin Elmer (MA, United States).

### Preparation of Plant Extracts

The leaves and roots of three varieties of *M. pumilum*, were collected from Hutan Gunung Bujang Melaka, Kampar, Perak, Malaysia. The plants were authenticated by Emeritus Professor Dato’ Dr. Abdul Latiff Mohamad and voucher specimens of MPA (UKMB 30006/SM 2622), MPP (UKMB 30007/SM s.n), and MPL (UKMB 30008/SM s.n) were deposited in the Herbarium of Universiti Kebangsaan Malaysia. Fresh plant materials were washed, garbled, air-dried and ground. Each dried powder was individually extracted with 80% ethanol in ratio of 1:10 by exhaustive maceration for 3 days at room temperature and was repeated for 10 times. The organic filtrate was collected and concentrated under reduced vacuum pressure to remove the residual organic solvent. The solvent-free extracts were combined and then freeze-dried to obtain crude. The crude extract was stored at 4°C until further use.

### Human Blood

Fresh venous blood was collected from healthy volunteers (*n* = 3, ≥18 years old) who fulfilled the following inclusion criteria of non-smoker, fasted overnight, and had not taken any medicines or supplements. The experimental protocol for *in vitro* cytokines and PGE_2_ assays was approved by the Human Ethical Committee of Universiti Kebangsaan Malaysia with approval number of UKM 1.5.3.5/244/NF- 040-2011 and UKM 1.5.3.5/244/NF- 016-2013, respectively. The protocol was in accordance to the principles outlined in the Declaration of Helsinki (World Medical Association [WMA], 2008).

### Animals

Male Sprague-Dawley rats (6–8 weeks old, 300–350 g) were obtained from Laboratory Animal Resource Unit, Universiti Kebangsaan Malaysia (LARU-UKM), Malaysia. The animals were housed in plastic cages and maintained on a 12 h/12 h light/dark cycle and the temperature and humidity were kept at 25 ± 2°C and 50%, respectively. They were fed with a commercial laboratory diet and allowed food and water *ad libitum* for the duration of the study. They were allowed one week to adapt to their environment before the experiment. All the procedures were carried out in accordance with A CIOMS Ethical Code for Animal Experimentation and approved by the Universiti Kebangsaan Malaysia-Animal Ethics Committee (UKMAEC) with approval number FF/2013/JAMIA/25-SEPT/537-OCT.2013-FEB.2015 (Howard-Jones, 1985).

### Anti-hyperuricemic Effect

#### *In vitro* Xanthine Oxidase Inhibition Assay

Xanthine oxidase (XO) inhibitory activity was measured using a slightly modifying spectrophotometric method as described by Hudaib et al. (2011), Noro et al. (1983), and Sweeney et al. (2001) using 96-well plates. A mixture consisted of 130 μL of phosphate buffer solution, 10 μL of working solution of extract (400 μg/mL) or controls (100 μg/mL of allopurinol or 0.5% of DMSO) and 10 μL of xanthine oxidase solution (0.2 units/mL) were pre-incubated at 25°C for 15 min. The reaction only took place when 100 μL xanthine solutions (0.15 mM) was added and incubated for 10 min at 25°C. Negative control (0.5% DMSO) in the absence of any inhibitor and positive control (allopurinol) were run simultaneously. Test extracts and allopurinol were dissolved in DMSO and the final concentration of DMSO was less than 0.5%. The blank of each sample was prepared in the same way, but in the absence of xanthine oxidase solution. The enzymatic conversion of xanthine to uric acid as a final product was measured at an absorbance of 295 nm using Multiskan^TM^ GO Microplate spectrophotometer. The percentage of XO inhibitory activity was calculated as Eq. 1:

(1) $$Percentageinhibition\left(\%\right)=\left[\frac{\left(A-B\right)-\left(C-D\right)}{\left(A-B\right)}\right]\times 100$$

where *A* is the enzyme activity without test extract; *B* is the control of *A* without test solution and enzyme, *C* and *D* are the activities of the test extract with and without enzyme, respectively. Using this method, the activity of six samples were screened and the active extracts with activity ≥50% were further assayed using varying concentrations ranging from 25 to 400 μg/mL to obtain its half maximal inhibitory concentration (IC_50_) values which calculated using GraphPad Prism^®^ (version 5.0, Sandiego, CA, United States).

Lineweaver-Burk plot was analyzed in order to determine the mode of enzyme inhibition of the most potent extract and allopurinol. The xanthine oxidase enzyme kinetic study was performed in a similar way to assay procedure for *in vitro* XO inhibitory with varying concentrations of xanthine substrate solution (150, 100, 50, 25 and 0 μM) (Umamaheswari et al., 2007). The Lineweaver-Burk plots were generated using GraphPad Prism^®^ (version 5.0, Sandiego, CA, United States).

#### *In vivo* Anti-hyperuricemic Effect

Animals were divided into six groups (*n* = 6).

Group I: normal control group.
Group II: hyperuricemic control group, received 250 mg/kg of potassium oxonate, intraperitoneally (i.p) on 1st, 7th, and 14th day as a negative control.
Group III: animals treated with 5 mg/kg of allopurinol, orally, for 14 days and received 250 mg/kg of potassium oxonate, i.p on 1st, 7th, and 14th day as a positive control.
Group IV: animals treated with 50 mg/kg of MPP leaves extract, orally, for 14 days and received 250 mg/kg of potassium oxonate, i.p on 1st, 7th, and 14th day.
Group V: animals treated with 100 mg/kg of MPP leaves extract, orally, for 14 days and received 250 mg/kg of potassium oxonate, i.p on 1st, 7th, and 14th day.
Group VI: animals treated with 200 mg/kg of MPP leaves extract, orally, for 14 days and received 250 mg/kg of potassium oxonate, i.p on 1st, 7th, and 14th day.

Potassium oxonate (125 mg/mL) dissolved in 0.9% saline solution was administered intraperitoneally 1 h before oral administration of test samples or allopurinol on the 1st, 7th, and 14th day of experiment. Samples (100 mg/mL) and allopurinol (10 mg/mL) were individually suspended homogeneously in 3% Tween 20. Animals were fasted 2 h before drug administration. Treatments were administered once a day by oral gavage for 14 consecutive days.

Rats were anesthetised 1 h after drug/extract administration on 1st, 7th and 14th days in order to collect blood from retro-orbital plexus. The blood was allowed to clot for approximately 1 h at room temperature and then centrifuged at 2500 × *g* for 10 min at 4°C. Serum was separated and stored at −20°C until assay for uric acid quantification. At the end of experiment, rats were sacrificed by cervical dislocation after blood collection and under mild anesthesia. The organs (liver, kidneys, spleen, heart and lung) were immediately excised, washed in cold saline (0.9%), dried with paper towel, weighed and rapidly stored at −80°C.

#### Uric Acid Assay

The serum uric acid levels were determined by enzymatic-colorimetric method using a uric acid assay kit (BioVision, Milpitas, CA, United States). The protocol was in accordance of kit manufacturer’s instruction. Briefly, in each well, 5 μL of serum, 45 μL of uric acid assay buffer and 50 μL reaction mixture (46 μL of uric acid buffer, 2 μL of uric acid probe and 2 μL of uric acid enzyme mixture) were mixed. The same relative volume was used for the calibration curve and blank. The reaction mixture was incubated for 30 min and the absorbance was measured using a microplate reader at a wavelength of 570 nm.

#### Liver Xanthine Oxidase Assay

Crude enzyme extract was prepared according to the method by Haidari et al. (2009). Briefly, 1 g of liver was homogenized in 5 mL of 80 mM cold sodium phosphate buffer (pH 7.4) and the homogenate was centrifuged at 3000 × *g* for 10 min at 4°C. Lipid layer was carefully removed and the supernatant was further centrifuged at 10,000 × *g* for 60 min at 4°C. The final supernatant was used for liver XO assay determined using the XO colorimetric assay kit from BioVision (Milpitas, CA, United States) and measured at a wavelength of 570 nm. One unit of XO is the amount of enzyme which catalyzes the oxidation of xanthine, yielding 1 μmol of uric acid and H_2_O_2_ per minute at 25°C. Protein concentration was determined spectrophotometrically based on the method of Bradford (1976), using bovine serum albumin as the standard.

### Anti-inflammatory Effects

#### *In vitro* Cytokines Assay

##### Cell preparation and viability test

The fresh blood collected from healthy volunteer was obtained in heparin-containing tube. Then, PBMCs were isolated by Lymphoprep gradient separation method as describe by us previously (Rahmi et al., 2017). Cell viability test was performed in a tissue culture 96-well microplate and determined by MTT assay (Rahmi et al., 2017). The PBMCs suspension (100 μL)were incubated with 100 μL of extracts (50 and 100 μg/mL) or dexamethasone (0.5 and 5 μg/mL) or complete medium with 0.5% DMSO (negative control) at 37°C with 5% CO_2_ for 27 h. The plates were incubated again for 4 h with 20 μL of MTT (5 mg/mL). The supernatant was carefully discarded and the formazan blue crystals produced by cells were dissolved in 100 μL of DMSO (100%) and the absorbance was measured at a wavelength of 570 nm.

##### Preparation of monosodium urate crystals

Monosodium urate (MSU) crystals were prepared according to previously described method with slight modification (Sabina et al., 2010). Briefly, 4 g of uric acid was dissolved and heated in 800 mL H_2_O with NaOH (9 mL/0.5 N); adjusted to pH 8.9 at 60°C by adding HCl, cooled for 3 days in cold room, then washed and dried. Then, the MSU crystals were sterilized by heating at 180°C for 2 h before experiments. Needle-shaped crystals were suspended in sterile saline to give a suspension of 200 μg/mL.

##### Determination of cytokine levels

The PBMCs (100 μL) were pre-incubated with equal volume of extracts or dexamethasone as a positive control or complete medium with 0.5% DMSO as a negative control for 3 h at 37°C in 5% CO_2_. After pre-incubation, cells were incubated with 20 μL of MSU crystal suspension (200 μg/mL) for 24 h. After incubation, cells were centrifuged for 10 min at 300 × *g* and 4°C. The supernatant was carefully transferred into a sterile tube and the concentration of cytokines in the supernatant was measured using appropriate ELISA kits for human. The cytokine secretion levels were compared with the negative control that was considered as 100% cytokine secretion. The percentage inhibition (% I) was calculated using Eq. 2:

(2) $$\%I=\left(1-\frac{\begin{array}{c} {}[ConcentrationofcytokineorPGE2\\ insampleorpositivecontrol] \end{array}}{\begin{array}{c} {}[concentrationofcytokineorPGE2\\ innegativecontrol] \end{array}}\right)\times 100\%$$

#### *In vitro* PGE_2_ Assay

##### Plasma preparation

Blood (1 mL) was obtained in heparin-containing tube and pre-incubated for 30 min in the presence or absence of extracts (50 μg/mL) or indomethacin (10 μg/mL) as a positive control or 0.5% DMSO in RIA buffer as negative control. Then, the mixture was incubated at 37°C containing 5% CO_2_ for 24 h with 10 μL MSU crystals suspension (200 μg/mL). The plasma was obtained by centrifugation at 3000 × *g* for 15 min at 4°C (Rahmi et al., 2017).

##### Radiommunoassay of plasma PGE_2_ secretion

The assay was carried out according to our method as previously reported (Rahmi et al., 2017). The plasma (100 μL) was added to anti-PGE2 (100 μL; diluted with ratio of 1:50,000) and [3H]-PGE2 (0.1 μCi/mL) then incubated at 4°C for 24 h. After incubation, 200 μL of dextran-coated charcoal was added into the mixture and incubated again for 10 min at 4°C. After centrifugation at 3,000 × *g* for 15 min at 4°C, 300 μL of supernatant was added to 3 mL liquid scintillation cocktail in Pico Pro Vial (Perkin Elmer, MA, United States). The radioactivity was measured using a liquid scintillation analyzer (Packard Tri-Carb, models B3110TR, Hamburg, Germany). The normalized percentage bound (%B/Bo) was then calculated using Eq. 3.

(3) $$\%\frac{B}{B\,o}=\left(\frac{B-N\,c}{B\,o-N\,c}\right)\times 100\%$$

The %B/Bo values were plotted using semi-logarithmic graph against the corresponding concentration of standard PGE_2_ in picogram (ρg). The %B/Bo values of serial dilutions of standard PGE2 with concentrations ranging from 2.45 to 400 ρg/0.1 mL were used to obtain a standard curve plot. The concentration of PGE_2_ (ρg/0.1 mL) in each sample were determined by interpolating the %B/Bo values. Percentage inhibition was calculated using Eq. 2.

#### *In vivo* Anti-inflammatory Assay

An experimental model of gouty inflammation using monosodium urate (MSU) crystals as inducer was used in order to evaluate the anti-inflammatory effect of MPP roots extract, as described previously (Zhang et al., 2012) with slightly modifications. Animals were divided into six groups (*n* = 6).

Group I: normal control group.
Group II: MSU control group (received 50 μL of MSU crystals suspension (100 mg/mL), intra-articular in the right knee joint on 11th day) as a negative control.
Group III: animals treated with 3 mg/kg of indomethacin, orally, for 14 days and received 50 μL of MSU crystals suspension (100 mg/mL), intra-articular in the right knee joint on 11th day as a positive control.
Group IV: animals treated with 50 mg/kg of MPP roots extract, orally, for 14 days and received 50 μL of MSU crystals suspension (100 mg/mL), intra-articular in the right knee joint on 11th day.
Group V: animals treated with 100 mg/kg of MPP roots extract, orally, for 14 days and received 50 μL of MSU crystals suspension (100 mg/mL), intra-articular in the right knee joint on 11th day.
Group VI: animals treated with 200 mg/kg of MPP roots extract, orally, for 14 days and received 50 μL of MSU crystals suspension (100 mg/mL), intra-articular in the right knee joint on 11th day.

Monosodium urate crystals (100 mg/mL) suspended in 0.9% sterile saline solution was administered intra-articularly in the right knee joint of each animal, except those of normal control group on the 11th day of experiment. Samples (100 mg/mL) and indomethacin (10 mg/mL) were individually suspended homogeneously in 3% Tween 20. Animals were fasted 2 h before drug administration. Treatments were administered once a day by oral gavage for 14 consecutive days. At the end of experiment, rats were sacrificed after anesthesia in order to collect synovial fluid. After skin shaving, a 27 G needle was inserted into the synovial cavity to inject 100 μL sterile saline, while the second needle was inserted next to the first needle to collect synovial fluid and immediately stored at −80°C until assay. The organs (liver, kidneys, spleen, heart and lung) were immediately excised, washed in cold saline (0.9%), dried with paper towel and weighed.

The inflammation was evaluated by measuring TNF-α, IL-1α, IL-1β, IL-6, and PGE_2_ in synovial fluid. TNF-α, IL-1α, IL-1β, and IL-6 were measured using a multi-cytokine bead array detection system (Procarta, eBioscience, United States) according to the manufacturer’s instruction. Meanwhile, PGE_2_ (R&D, United States) were measured using single ELISA assay according to the manufacturer’s instruction.

### High Performance Liquid Chromatographic (HPLC) Analysis

Phytochemical analysis was performed for the most active extracts from *in vitro* and *in vivo* assays, i.e., leaf and root extracts of MPP, using RP-HPLC based on the method described by Bae et al. (2012) to quantify the amount of reference standards (i.e., kaempferol, myricetin and quercetin).

HPLC analysis was performed using a Waters system (Ireland, Dublin) with C-18 column (250 mm × 4.6 mm i.d., 5 μm), photodiode array detector at a wavelength of 360 nm and flow rate of 0.6 mL/min. The mobile phase consisted of methanol (A) and acidified water with 0.1% orthophosphoric acid (B) and eluted by a linear gradient of 40–100% A (0–10 min), an isocratic composition of 100% A (12–15 min), and a linear gradient of 100–40% A (15–20 min). The column was equilibrated for 20 min before next injection. Injection volume of each solution was 10 μL. The RP-HPLC method was validated based on the determination of linearity, precision, limits of quantification (LOQ), and detection (LOD) for three detected reference standards, i.e., quercetin, myricetin and kaempferol.

The extracts of MPP leaves and roots (100 mg/mL) and a mixture of reference standards of kaempferol, myricetin and quercetin (1 mg/mL) were prepared in HPLC-grade MeOH, sonicated for 5 min and filtered using 0.45 μm membrane prior to analysis using the validated HPLC conditions. Stock solution of mixture of reference standards was further diluted into a series of two-fold dilutions (62.5, 125, 250, 500, and 1000 μg/mL). The calibration curve was plotted with five concentrations of reference standards solution *versus* the areas under the peaks. The standard curve equation obtained from reference standards was used to quantify the concentration of flavonoids in extracts.

### Statistical Analysis

All data were analyzed using GraphPad Prism 5 software. Each experiment was carried out in triplicate (*n* = 3) and the data presented as mean ± standard error of mean (SEM). The IC_50_ values were calculated with a non-linear regression analysis using GraphPad Prism 5 software. The values were obtained from at least three determinations (*n* = 3). Data were statistically analyzed using one-way analysis of variance (ANOVA) and *post hoc* Tukey’s test for multiple comparisons and *p* ≤ 0.05 was considered to be statistically significant. Pearson’s correlation coefficient was used to analyze the degree of association between xanthine oxidase activities and uric acid levels *in vivo*.

## Results

### Anti-hyperuricemic Effect

#### *In vitro* Inhibition of Xanthine Oxidase Activity by *Marantodes pumilum* Extracts

In this study, the highest inhibition of XO activity was found from MPP leaves extract (400 μg/mL) with value of 87%. All extracts showed a lower activity compared to allopurinol as positive control (*p* ≤ 0.01). Allopurinol (100 μg/mL) strongly attenuated XO activity with percentage of inhibition value of 99.8% as shown in Table 1. All leaf extracts showed inhibition of more than 50%, whilst only MPP roots gave more than 50% of inhibition. Furthermore, the IC_50_ values were determined for those active extracts and all active extracts were found to possess a dose-dependent XO inhibitory effect as shown in Table 1.

**TABLE 1:** Xanthine oxidase inhibitory activity and IC_50_ values of extracts of *Marantodes pumilum* varieties.

**Species**	**Plant part**	**% Inhibition**	**IC_50_ (μg/mL)**
*M. pumilum* var. a*lata*	Roots	43.6 ± 1.2	−
	Leaves	58.1 ± 0.4	263.8 ± 9.5
*M. pumilum* var. *pumila*	Roots	52.5 ± 0.4	379.6 ± 4.0
	Leaves	86.9 ± 4.8	130.5 ± 5.3
*M. pumilum* var. *lanceolata*	Roots	47.8 ± 0.9	−
	Leaves	72.4 ± 0.3	165.1 ± 1.2
Allopurinol	−	99.8 ± 0.1	0.13 ± 0.0

*Data are presented as mean ± SEM (*n* = 3). Concentration of extracts was 400 μg/mL, whilst allopurinol was 100 μg/mL. (−) = not determined as none of the tested concentration exceeded 50% of inhibition. % inhibition >2.5% was significant at *p* ≤ 0.05 when compared with negative control. All% inhibition values of extracts were statistically different compared with allopurinol (*p* ≤ 0.01). All IC_50_ values of extracts were statistically different compared with allopurinol (*p* ≤ 0.001).*

The results revealed that MPP leave extract possessed the strongest XO inhibitory activity with IC_50_ value of 130.5 μg/mL. However, all extracts possessed significantly lower activity compared to allopurinol with IC_50_ value of 0.13 μg/mL (*p* ≤ 0.001).

**FIGURE 1:**
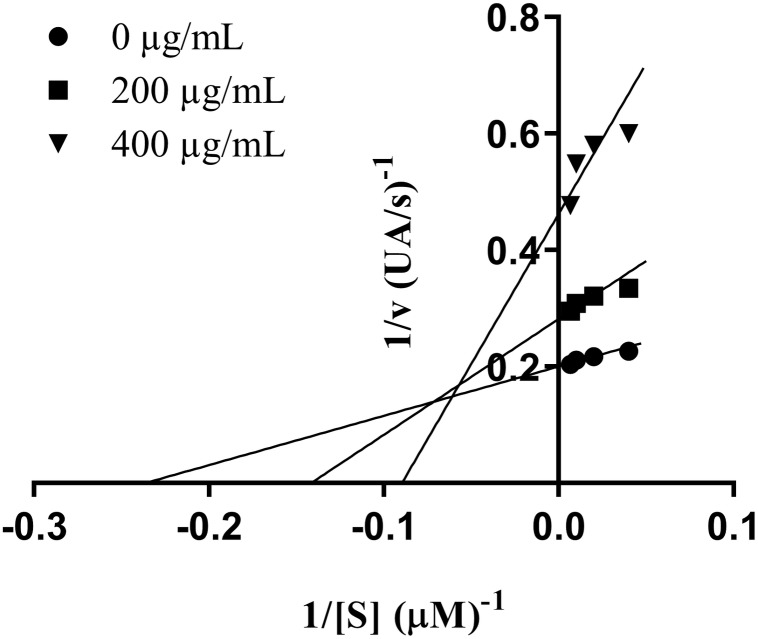
Lineweaver–Burk plot of inhibition of xanthine oxidase by *Marantodes pumilum* var. *pumila* leaf extract. Notes: • (0 μg/mL); ■ (200 μg/mL); ▼ (400 μg/mL).

Type of inhibitory activity of XO by MPP leaf extract was established from Lineweaver-Burk plots (Figure 1). The Michaelis-Menten constant (Km) increased from 4.2 to 11.2 mM, whereas maximum velocity (Vmax) of the reaction decrease from 5.0 to 2.2 mM/min. Therefore, the extract seems to induce a mixed type of inhibition.

#### Effect of *Marantodes pumilum* var. *pumila* Leaf Extract on Serum Uric Acid Levels in Hyperuricemic Rats

In this study, the baseline values of serum uric acid levels of each group, measured before starting MPP leaf extract and potassium oxonate administration (Day 0), ranged from 1.6 to 1.7 mg/dL as shown in Table 2. Treatment of hyperuricemic rats with MPP leaf extract at doses of 50, 100 and 200 mg/kg/day significantly reduced serum uric acid levels when compared to hyperuricemic control group (*p* ≤ 0.05). However, only treatment with 200 mg/kg/day of extract was able to reduce serum uric acid levels to the baseline value after 14 days of experiment (*p* > 0.05). Allopurinol (5 mg/kg) reduced serum uric acid levels compared to hyperuricemic control group (*p* ≤ 0.05). This reduction was observed even after 1 day of treatment with allopurinol and the normal serum uric acid levels were maintained throughout the 14 days of experiment. The data showed that MPP leaf extract elicited a slower onset of action compared to allopurinol. However, there was no significant difference between the effect of allopurinol and extract at the dose of 200 mg/kg at day 14 (*p* > 0.05). This demonstrated that the effectiveness of extract at the dose of 200 mg/kg was comparable to allopurinol after 14 days of treatment.

**TABLE 2:** Effect of *Marantodes pumilum* var. *pumila* leaf extract on serum uric acid levels in rats.

**Treatment**	**Dose (mg/kg)**	**Serum uric acid levels ± SEM (mg/dL)**			
		**Day 0**	**1st day**	**7th day**	**14th day**
Normal	−	1.6 ± 0.1	1.7 ± 0.1^b^	1.7 ± 0.1^b^	1.7 ± 0.0^b^
Hyperuricemic control	−	1.7 ± 0.2	3.3 ± 0.1^a^	3.2 ± 0.1^a^	3.2 ± 0.2^a^
Allopurinol	5	1.6 ± 0.1	1.6 ± 0.1^b^	1.6 ± 0.0^b^	1.4 ± 0.1^b^
Extract	50	1.6 ± 0.1	2.9 ± 0.2^a,b^	2.5 ± 0.1^a,b^	2.1 ± 0.1^a,b^
	100	1.7 ± 0.1	2.8 ± 0.1^a,b^	2.0 ± 0.0^a,b^	1.7 ± 0.1^a,b^
	200	1.7 ± 0.1	2.7 ± 0.1^a,b^	1.7 ± 0.1^a,b^	1.6 ± 0.0^b,c^

*Data are presented as mean ± SEM (*n* = 6). Data were analyzed using one-way ANOVA followed by *post hoc* Tukey. ^*a*^Significantly different compared to normal group (*p* ≤ 0.05). ^*b*^Significantly different compared to hyperuricemic group (*p* ≤ 0.05). ^*c*^Not significantly different compared to allopurinol group (*p* > 0.05).*

#### Effect of *Marantodes pumilum* var. *pumila* Leaf Extract on Xanthine Oxidase Activity in Hyperuriccemic Rats Liver

Liver XO inhibitory activity in rats was evaluated in order to further investigate the anti-hyperuricemic effect of the extract. The results revealed that potassium oxonate, beside producing hyperuricemia, cause a slight but significant induction of liver XO activity compared to normal control group (*p* ≤ 0.05) as shown in Table 3. Treatment with MPP leaf extract at the dose of 200 mg/kg/day was able to inhibit liver XO activity by 25%, whilst that of allopurinol inhibited XO activity by 45%. It showed that the inhibitory effect of allopurinol on XO activity was significantly higher than MPP leaf extract even at highest dosage of extract (*p* ≤ 0.05).

**TABLE 3:** Effect of *Marantodes pumilum* var. *pumila* leaf extract on xanthine oxidase activity in rat’s liver.

**Treatment**	**XO activity ± SEM (nmole uric acid/min per mg protein)**	**% Inhibition**
Normal	2.2 ± 0.1^b,c^	−
Hyperuricemic	2.8 ± 0.1^a,c^	−
Allopurinol (5 mg/kg)	1.5 ± 0.0^a,b^	44.8 ± 0.2
Extract (50 mg/kg)	2.3 ± 0.1^b,c^	18.9 ± 0.5^c^
Extract (100 mg/kg)	2.2 ± 0.1^b,c^	20.9 ± 0.5^c^
Extract (200 mg/kg)	2.1 ± 0.1^b,c^	25.3 ± 0.2^c^

*Data are presented as mean ± SEM (*n* = 6). Data were analyzed using one-way ANOVA followed by *post hoc* Tukey. ^*a*^Significantly different compared to normal group (*p* ≤ 0.05). ^*b*^Significantly different compared to hyperuricemia group (*p* ≤ 0.05). ^*c*^Significantly different compaped to allopurinol (*p* ≤ 0.05).*

**FIGURE 2:**
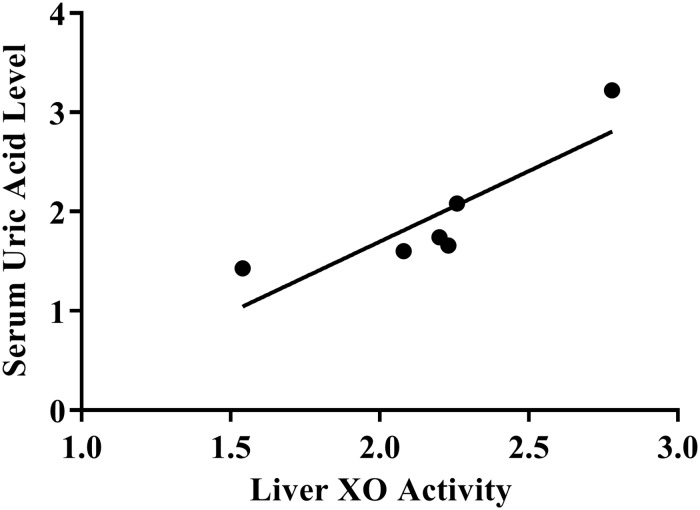
Scatterplot of correlation between xanthine oxidase activity and serum uric acid level.

Treatment with MPP leaf extract showed a dose-dependent effect on both serum uric acid levels and liver XO inhibitory activity (Figure 2). A Pearson’s correlation coefficient was performed to assess the relationship between liver XO activity and serum uric acid levels. The result showed that there was a positive correlation between the two variables (*r* = 0.87, *n* = 6, *p* = 0.03).

### Anti-inflammatory Effect

#### Effect of *Marantodes pumilum* Extracts on MSU-Stimulated Cytokine Secretion

##### Cell viability of human peripheral blood mononuclear cells

MTT assay method was used to assess the cell viability of PBMCs to *M. pumilum* extracts at 50 and 100 μg/mL. Per cent (%) viability of all *M. pumilum* extracts at concentration of 50 μg/mL and dexamethasone at 5 μg/mL was greater than 90% after 27 h of exposure (Supplementary Figure S1), indicating that extracts (50 μg/mL) and dexamethasone (5 μg/mL) were non-toxic against PBMCs. Thus, the respective concentrations were used as the highest concentration in this experiment.

##### Inhibitory effect of marantodes pumilum extracts on cytokine secretion in MSU-treated human peripheral blood mononuclear cells

The inhibitory activity on the secretion of five cytokines (i.e., IL-1α, IL-1β, IL-6, IL-8, and TNF- α) of *M. pumilum* extracts at a concentration of 50 μg/mL on PBMCs was shown in Table 4. Out of six extracts tested, three extracts (i.e., MPP roots, MPL roots and leaves) were found to actively inhibit (>50% inhibition) cytokine secretion as compared with negative control. MPP roots possessed the highest inhibition against secretion of four cytokines, i.e., IL-1α, IL-1β, IL-8, and TNF-α. Meanwhile, MPL roots actively inhibited the secretion of three cytokines, that were, IL-1α, IL-1β, and TNF-α, whilst that of the leaf part inhibited only TNF-α. Dexamethasone (5 μg/mL) as a positive control successfully inhibited secretion of five cytokines, i.e., IL-1α, IL-1β, IL-6, IL-8, and TNF-α. Statistical analysis revealed that the inhibitory activities of all active extracts against IL-1β and IL-8 were comparable (*p* > 0.05) to dexamethasone. Only MPP roots showed significantly higher (*p* ≤ 0.05) inhibitory activity against TNF-α compared to dexamethasone, whilst that of the other active extracts were comparable (*p* > 0.05) to dexamethasone. However, all active extracts showed significantly lower (*p* ≤ 0.05) inhibitory activity against IL-1α than dexamethasone.

**TABLE 4:** Percentage of inhibition of extracts of *Marantodes pumilum* varieties (50 μg/mL) on cytokine secretion in MSU-stimulated human PBMCs.

**Species**	**Plant part**	**% Inhibition**				
		**IL-1α**	**IL-1β**	**IL-6**	**IL-8**	**TNF- α**
*M. pumilum* var. *alata*	Roots	26.5 ± 4.0	28.1 ± 4.1	19.3 ± 6.6	25.1 ± 6.8	15.0 ± 12.5
	Leaves	22.6 ± 0.7	36.1 ± 4.6	27.0 ± 6.0	25.1 ± 6.8	12.6 ± 12.1
*M. pumilum* var. *pumila*	Roots	65.3 ± 2.3	65.3 ± 2.3*	41.0 ± 4.8	58.1 ± 10.3*	84.5 ± 27.6
	Leaves	38.5 ± 4.1	11.6 ± 5.8	ND	43.9 ± 8.6	41.5 ± 13.9
*M. pumilum* var. *lanceolata*	Roots	52.5 ± 3.1	53.7 ± 3.1*	44.7 ± 4.5	46.6 ± 10.3	75.0 ± 15.0
	Leaves	47.2 ± 3.5	47.9 ± 3.4	35.8 ± 5.2	42.5 ± 13.7	58.2 ± 20.2*
Dexamethasone		87.4 ± 0.8	55.0 ± 3.0	66.6 ± 2.7	60.5 ± 1.7	67.5 ± 2.4

Data are presented as mean ± SEM (*n* = 3). Data were analyzed using one-way ANOVA followed by *post hoc* Tukey. ND = inhibition was not detected. Percentage inhibition >2.5% was significant at *p5* ≤ 0.05 when compared with negative control. *p ≥ 0.05 was considered not significantly different compared to dexamethasone (5 μg/mL).

Determination of IC_50_ value of active extracts demonstrated that the inhibitory activity was in a concentration dependent manner. The IC_50_ values for all active extracts were significantly higher than dexamethasone as positive control (*p* ≤ 0.001). The results showed that extract of MPP roots possessed the lowest IC_50_ values for IL-1α (35.71 μg/mL), IL-1β (25.06 μg/mL), and IL-8 (38.4 μg/mL), whilst MPL roots showed the lowest IC_50_ value for TNF-α (15.38 μg/mL) as seen in Table 5.

**TABLE 5:** IC_50_ values (μg/mL) of *Maratodes pumilum* extracts on cytokine secretion in MSU-induced human PBMCs.

**Species**	**Plant part**	**IC_50_ (μg/mL)**				
		**IL-1α**	**IL-1β**	**IL-6**	**IL-8**	**TNF-α**
*M. pumilum* var. *pumila*	Roots	35.7 ± 2.1	25.01 ± 1.6	−	38.4 ± 6.5	17.7 ± 1.2
*M. pumilum* var. *lanceolata*	Roots	46.0 ± 5.2	44.7 ± 5.5	−	−	15.4 ± 0.8
	Leaves	−	−	−	−	35.7 ± 1.8
Dexamethasone		0.02 ± 0.0	0.7 ± 0.2	0.5 ± 0.0	0.7 ± 0.1	0.1 ± 0.0

*Data are presented as mean ± SEM (*n* = 3). Data were analyzed using one-way ANOVA followed by *post hoc* Tukey. (−) = not determined as none of tested concentration exceeded 50% inhibition. All IC_50_ values of extracts were statistically different compared to dexamethasone (*p* ≤ 0.001).*

#### Effect of *Marantodes pumilum* Extracts on Plasma Prostaglandin E_2_ Secretion

Out of six extracts (50 μg/mL), roots and leaves of MPP were found to actively inhibit PGE_2_ secretion (Table 6). However, all active extracts gave significantly lower activity (*p* ≤ 0.05) compared to indomethacin (10 μg/mL). The result showed that out of the two active extracts, the IC_50_ value of extracts of MPP leaves (IC_50_ 42.33 μg/mL) was found to be more potent than the root part (IC_50_ 45.52 μg/mL). However, the IC_50_ values of active extracts were significantly higher (*p* ≤ 0.001) than indomethacin with IC_50_ value of 0.35 μg/mL.

**TABLE 6:** Inhibitory activities of extracts of *Marantodes pumilum varieties*extracts (50 μg/mL) and IC_50_ values (μg/mL) on PGE_2_ production in MSU-stimulated human whole blood.

**Species**	**Plant Part**	**% inhibition**	**IC_50_**
*M. pumilum* var. *alata*	Roots	30.7 ± 0.5	–
	Leaves	13.1 ± 5.8	–
*M. pumilum* var. *pumila*	Roots	59.7 ± 1.3	45.5 ± 0.6
	Leaves	58.4 ± 1.3	42.3 ± 0.6
*M. pumilum* var. *lanceolata*	Roots	40.3 ± 0.2	–
	Leaves	40.8 ± 0.3	–
Indometachin		97.4 ± 0.7	0.4 ± 0.5
Negative control		0	–

Data are presented as mean ± SEM (*n* = 3). Data were analyzed by using one-way ANOVA followed by *post hoc* Tukey. ND = inhibition was not detected. (−) = not determined as none of tested concentration exceeded 50% inhibition. Percentage inhibition >2.5% was significant at *p* ≤ 0.05 when compared with negative control. *p ≥ 0.05 was considered not significant compared with indomethacin (positive control).

#### Effect of *Marantodes pumilum* var. *pumila* Roots Extracts on Cytokine and Prostaglandin E_2_ in Rat Synovial Fluid

**FIGURE 3:**
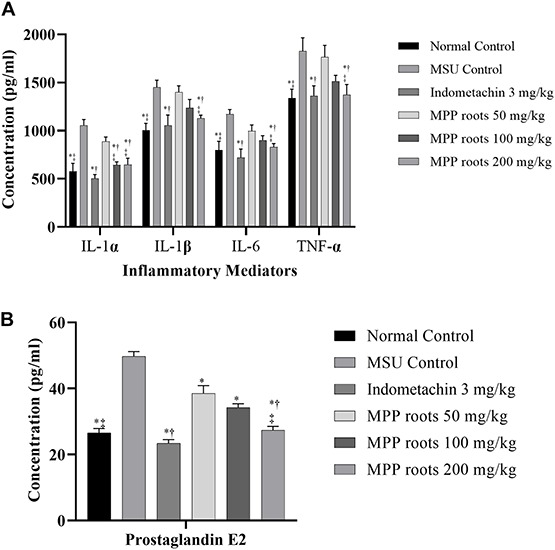
Effect of *Marantodes pumilum* var. *pumila* root extract on inflammatory mediators secretion in monosodium urate crystal-induce inflammation on rat knee joint. **(A)** Cytokines, **(B)** prostaglandin E_2_. Data are presented as mean ± SEM of 6 animals. *Significantly different compared to MSU control group (*p* ≤ 0.05). †not significantly different compared to normal control group (*p* > 0.05). ‡not significantly different compared to indomethacin group (*p* > 0.05).

Articular inflammation was quantified by examining the changes in markers attenuated by the treatment. Figure 3 represents the activity of extract of MPP roots on inflammatory mediators in the synovial fluid of experimental animals. The results showed higher levels of IL-1α, IL-1β, IL-6, TNF-α, and PGE_2_ in synovial fluid of MSU control rats when compared with normal control rats (*p* ≤ 0.05). However, treatment with the extract reduced levels of these inflammatory mediators in a dose-dependent manner. Both indomethacin and 200 mg/kg of MPP roots extract significantly reduced all cytokines and PGE_2_ to similar levels. It demonstrated that treatment with extract of MPP roots at dose of 200 mg/kg for 14 days was as effective as indomethacin at 3 mg/kg.

#### Body and Organ Weight Observation

**FIGURE 4:**
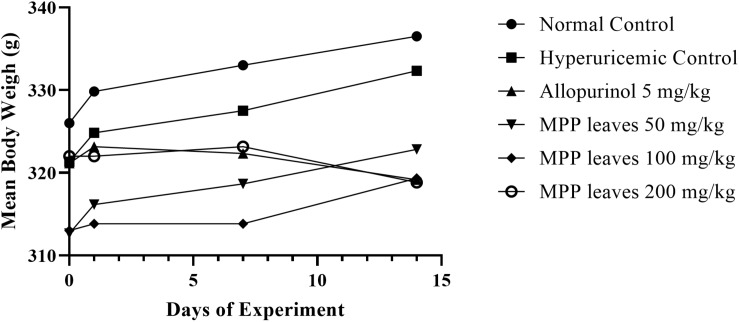
Body weight changes in experimental animals monitored before intervention (Day 0) and on Day 1, 7, and 14. Data were analyzed by using one-way ANOVA and followed by *post hoc* Tukey. No significant difference between Days 1, 7, and 14 vs. respected Day 0 (*p* > 0.05).

**FIGURE 5:**
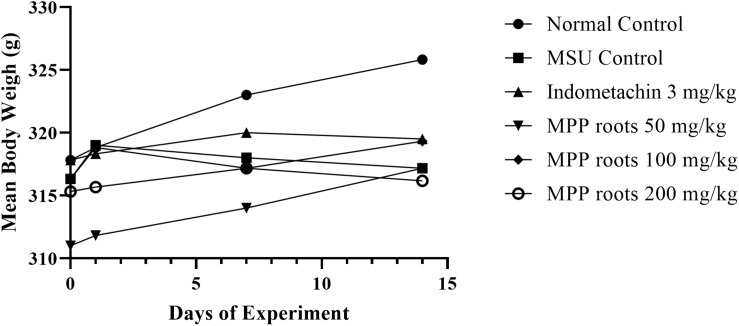
Body weight changes in experimental animals monitored before drug intervention (Day 0) and on Days 1, 7, and 14. Data were analyzed by using one-way ANOVA and followed by *post hoc* Tukey. No significant difference between Days 1, 7, and 14 vs. respected Day 0 (*p* > 0.05).

Body weight did not show significant differences among groups during the 14 experimental days (Figures 4, 5) although a slight non-significant body weight decrease occurred with allopurinol and 200 mg/kg of extract. Also, proportional weight of liver, kidney, spleen, heart and lung did not differ among groups (Tables 7, 8).

**TABLE 7:** Index organ of rats after 14 days of experiment using *Marantodes pumilum* var. *pumila* leaf extract.

**Group**	**Index organ (%)**					
	**Liver**	**Kidney (L)**	**Kidney (R)**	**Spleen**	**Heart**	**Lung**
Normal	4.2 ± 0.1	0.4 ± 0.01	0.3 ± 0.01	0.3 ± 0.01	0.4 ± 0.02	0.7 ± 0.02
Hyperuricemic	4.3 ± 0.11	0.3 ± 0.02	0.4 ± 0.02	0.4 ± 0.02	0.4 ± 0.01	0.7 ± 0.05
Allopurinol (5 mg/kg)	4.1 ± 0.29	0.4 ± 0.01	0.4 ± 0.01	0.4 ± 0.01	0.4 ± 0.01	0.7 ± 0.06
Extract (50 mg/kg)	4.1 ± 0.1	0.3 ± 0.01	0.4 ± 0.01	0.4 ± 0.01	0.4 ± 0.01	0.7 ± 0.02
Extract (100 mg/kg)	4.1 ± 0.09	0.4 ± 0.01	0.4 ± 0.02	0.3 ± 0.01	0.4 ± 0.01	0.7 ± 0.02
Extract (200 mg/kg)	4.1 ± 0.03	0.3 ± 0.01	0.3 ± 0.01	0.3 ± 0.01	0.3 ± 0.01	0.7 ± 0.01

*Data are presented as mean ± SEM (*n* = 6). Data were analyzed using one-way ANOVA followed by *post hoc* Tukey. Index organ was calculated as (organ weight/body weight) x 100%. No significant differences between treatment groups vs. normal group (*p* > 0.05).*

**TABLE 8:** Index organ of rats after 14 days of experiment using *Marantodes pumilum* var. *pumila* root extract.

**Group**	**Relative organ weight (%)**					
	**Liver**	**Kidney (L)**	**Kidney (R)**	**Spleen**	**Heart**	**Lung**
Normal	4.2 ± 0.10	0.4 ± 0.01	0.3 ± 0.01	0.3 ± 0.01	0.4 ± 0.02	0.7 ± 0.02
MSU control	4.3 ± 0.11	0.3 ± 0.02	0.4 ± 0.02	0.4 ± 0.02	0.36 ± 0.01	0.7 ± 0.05
Indomethacin (3 mg/kg)	4.1 ± 0.29	0.4 ± 0.01	0.4 ± 0.01	0.4 ± 0.01	0.4 ± 0.01	0.7 ± 0.06
Extract (50 mg/kg)	4.1 ± 0.1	0.3 ± 0.01	0.4 ± 0.01	0.4 ± 0.01	0.4 ± 0.01	0.7 ± 0.02
Extract (100 mg/kg)	4.1 ± 0.09	0.4 ± 0.01	0.4 ± 0.02	0.3 ± 0.01	0.4 ± 0.01	0.7 ± 0.02
Extract (200 mg/kg)	4.1 ± 0.03	0.33 ± 0.01	0.3 ± 0.01	0.3 ± 0.01	0.3 ± 0.01	0.7 ± 0.01

*Data are presented as mean ± SEM (*n* = 6). Data were analyzed using one-way ANOVA followed by *post hoc* Tukey. Relative organ weight was calculated as (organ weight/body weight) × 100%. No significant differences between treatment group vs. normal group.*

### HPLC Analysis

Based on the results obtained for linearity, LOD and LOQ, the method was giving a linear response in the selected range. LOD was acceptable and the method was able to detect and quantify the analyte below the minimum concentration of flavonoids used in the calibration curve (Ravichandran et al., 2010). The precision of HPLC method regarding repeatability was acceptable as indicated by the %RSD not more than 5% of peak area and retention time within intraday and interday assays (Crowther, 2001; Shabir, 2004).

**FIGURE 6:**
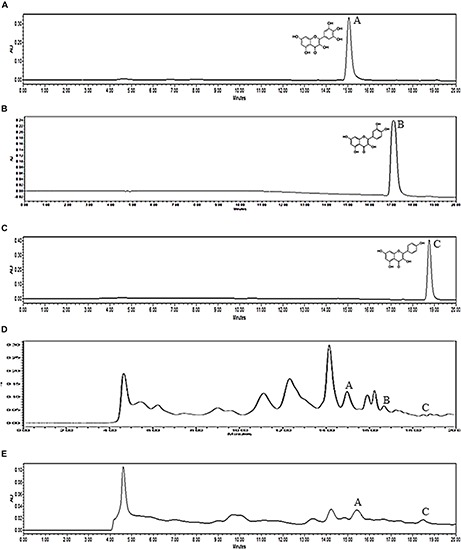
HPLC chromatograms of myricetin **(A)**, quercetin **(B)**, kaempferol **(C)** standards and ethanol (80%) extracts of *Marantodes pumilum* var. *pumila* leaves **(D)** and roots **(E)** detected at 360 nm. Retention time for myricetin (1): 14.95 min, quercetin (2): 17.13 min, kaempferol (3): 18.72 min.

In this study, three flavonoids, reportedly present in MPP roots and leaves, have been identified using HPLC, i.e., myricetin, quercetin and kaempferol, with retention time at 14.95, 17.13, and 18.72 min, respectively (Figure 6). The peaks were compared with reference standards. The amount of flavonoids in MPP leaves and roots are shown as Table 9. Both extracts were found to contain myricetin and kaempferol. However, only leaf extract of MPP was found to contain quercetin. Myricetin was detected at a higher concentration than the other detectable flavonoids with values of 0.19 and 0.63 mg/g for root and leaf extracts, respectively. Overall, the leaf extract was found to contain more flavonoids than root extract.

**TABLE 9:** Validation data of HPLC method for myricetin, quercetin and kaempferol standards.

**Standard**	**Conc. Range (μg/mL)**	Intra-Day Precision (% RSD)*						Inter-Day Precision (% RSD)**		**Linear Regression Equation/R^2^**	**LOD (ng/mL)**	**LOQ (ng/mL)**	Conc. of compound in crude extracts (mg/g)*	
		**Day 1**		**Day 2**		**Day 3**		**RT**	**Area**				**MPP leaves**	**MPP roots**
		**RT**	**Area**	**RT**	**Area**	**RT**	**Area**							
Myricetin	62.5	1.21	3.44	1.20	2.86	1.04	2.32	0.72	1.85	*y* = 50290 *x* − 58345	1.77	5.35	0.63 ± 0.03	0.19 ± 0.01
	125	0.95	1.24	1.06	1.26	0.64	0.52	0.42	0.57	(*R*^2^ = 0.998)				
	250	0.97	0.83	0.61	0.87	0.62	1.23	0.13	0.15					
	500	0.78	0.43	0.28	0.33	0.12	0.60	0.42	0.16					
	1000	0.30	0.18	0.41	0.20	0.64	0.13	0.22	0.28					
Quercetin	62.5	0.18	1.58	0.08	1.84	0.18	1.77	0.46	2.72	*y* = 50964 *x* − 424906	1.61	4.88	0.20 ± 0.01	ND
	125	0.07	1.70	0.18	1.02	0.34	1.89	0.42	4.99	(*R*^2^ = 0.998)				
	250	0.07	0.72	0.14	0.30	0.39	0.39	0.41	0.58					
	500	1.03	0.63	0.04	0.60	0.31	0.62	0.69	0.54					
	1000	0.04	0.68	0.26	0.23	0.04	0.51	0.37	0.29					
Kaempferol	62.5	0.60	2.41	0.42	1.98	0.07	1.09	0.70	1.75	*y* = 51357 *x* − 95293	5.21	15.77	0.04 ± 0.00	0.04 ± 0.001
	125	0.05	1.39	0.12	1.90	0.10	1.09	0.07	1.28	(*R*^2^ = 0.992)				
	250	0.20	1.10	0.12	1.58	0.16	0.74	0.09	0.27					
	500	0.08	0.29	0.20	0.56	0.43	0.25	0.18	0.34					
	1000	0.16	1.28	0.12	1.82	0.12	1.83	0.10	0.99					

*n = 3, **n = 9. ND = not detected.

## Discussion

Gout is caused by increased level of uric acid in blood (hyperuricemia) due to metabolism disorders of purine. Uric acid is the end product ofseveral deamination and oxidation processes of adenin and guanine, with xanthine oxidase (XO) widely distributed in the body, including liver, intestine, kidney, lungs, myocardium, brain, plasma and other tissues (Borges et al., 2002; Choi et al., 2005). A value of uric acid in blood greater than 7 mg/dL is consider an indicator of supersaturation of the body fluid. The persistent supersaturated condition of body fluid leads to crystallization of monosodium urate (MSU) and deposition in the joints, tendon and tissues, leading to transient and recurrent attacks of acute inflammation (Rosenberg, 2010; Chen et al., 2011).

In the present study, extract of MPP leaves showed anti-hyperuricemic effects *in vivo* and XO inhibitory activity *in vitro*. *In vitro* study showed that MPP leaves inhibited XO, although it was ten-times less potent than allopurinol. Phenolics, such as flavonoids, have been reported to possess XO inhibitory activity (Kostic et al., 2015). The structure-activity relationship of flavonoids as XO inhibitor has been reported. The presence the C-5 and the C-7 hydroxyl groups and the double bond between C-2 and C-3 were essential for a potent XO inhibitory activity (Cos et al., 1998). On the other hand, the derivatization of C-7 as an *O*-glycoside or a methyl ether and the glycosylation of C-3 hydroxyl group caused a decrease of XO inhibitory activity of flavonoids. *M. pumilum* has been reported to contain various flavonoids including quercetin, myricetin, kaempferol, and naringin that have been reported to possess XO inhibitory activity (Chua et al., 2011; Karimi et al., 2011b; Lin et al., 2015). However, most of the flavonoids in *M. pumilum* were found as flavonoid glycoside that was reported to possess lower XO inhibitory activity compared to flavonoid aglycones (Cos et al., 1998; Lin et al., 2002; Chua et al., 2011). Moreover, the presence of numerous phytochemical compounds in *M. pumilum* could lead to positive interactions, such as potentiation, synergism and complementary activities as XO inhibitors and anti-inflammatory agents or negative interactions between components, thus could decrease the activity of active compounds in the extracts. For example, the reduced forms of phytophenolics are powerful antioxidants equivalent to ascorbate, in contrast, the phenoxyl radical produced through antioxidative reactions and in lignin biosynthesis, is a potential prooxidant (Sakihama et al., 2002). Furthermore, the Lineweaver-Burk plot revealed that MPP leaves possessed a mixed-type of inhibition. Numerous plant extracts that have been reported to possess XO inhibitory activity showed a mixed-type of inhibition, such as aqueous fraction of ethyl acetate extract of *Fraxinus angustifolia* barks, aqueous fractions of hexane and chloroform extracts of *Pistacia lentiscus* leaves (Berboucha et al., 2010), chloroform fraction of MeOH (70%) extract of *Erythrina stricta* leaves (Umamaheswari et al., 2009), methanol extracts of *Cinnamomum cassia* twigs, *Chrysanthemum indicum* flowers and *Lycopus europaeus* leaves (Kong et al., 2000). Similar to *M. pumilum*, many of these plants contained phenolics and flavonoids. Although phenolic and flavonoid compounds were most likely reported to possess a competitive-type inhibition of XO, the bulky substituent groups of the phenolic and flavonoid structure were found to affect the activity (Lin et al., 2015). Thus, the flavonoid glycosides in *M. pumilum* might be responsible for the mixed-type XO inhibitory activity of this extract.

*In vivo* study revealed that despite of the positive correlation between liver XO inhibitory activity and serum uric acid levels in animal models, the effect of extract at the dose of 200 mg/kg/day on serum uric acid levels was comparable (*p* > 0.05) to allopurinol, but the effect on liver XO inhibitory activity was significantly lower than allopurinol (*p* ≤ 0.05). It has to be considered that the reduction of serum uric acid levels could be accomplished mainly through other mechanism of action such as uricosuric activity probably by modulating human urate transporter 1 (URAT1), glucose transporter 9 (GLUT9) and organic anion transporter 1(OAT1) (Burns and Wortmann, 2012; Shi et al., 2012). Flavonoids, such as quercetin, rutin, and kaempferol which present in *M. pumilum*, were reported to possess not only XO inhibitory activity but also uricosuric effects (Shi et al., 2012). Hence, it is important to further investigate the uricosuric effect of *M. pumilum*.

From this study, only MPP root, MPL root and leaf extracts gave inhibitory activities in MSU-induced inflammation of cytokines and PGE2 secretion. However, the IC_50_ values were significantly lower than dexamethasone. Out of the three active extracts, two were root part while only one was leaf part of *M. pumilum* varieties. It demonstrated that the root part maybe contained more active components compared to the leaf part and it affected their biological activity.

The experiment using MSU-induced inflammation has been used to simulate acute attack of gout occur due to uric acid crystallization. The intraarticular injection of MSU crystals in rat’s joint resembles gouty inflammation condition, that causes a painful response which similar to acute gout flare (Gentle, 1997). MSU crystals can interact with all of the major synovial cell types to produce a variety of inflammatory mediators, including pro-inflammatory cytokines and PGE_2_. These cytokines and PGE_2_ biologically act to cause the clinical features of inflammation include severe pain, edema, and erythema in the joint (Pauliot et al., 1998).

The anti-inflammatory effect *in vivo* study revealed that MPP root extracts reduced levels of IL-1α, IL-1β, IL-6, TNF-α, and PGE2 secretion on MSU-induced inflammation in rat’s synovial fluid. This findings was in line with a study by Inokuchi et al. (2006) that reported that the presence of MSU crystals in gouty inflammation activated macrophages, monocytes, synoviocytes, platelets, and neutrophils, hence causes an inflammatory response which is hallmarked by secretion of various inflammatory mediators, including IL-1, IL-6, IL-8, TNF-α, and PGE_2_.

The amounts of flavonoids in the MPP leaf extract was superior that the MPP root extract. Interestingly, MPP leaf extract only seem to possess higher xanthine oxidase inhibitory activity, thus it proceeded to *in vivo* anti-hyperuricemic assay. Meanwhile, the MPP root extract was proceeded to *in vivo* anti-inflammatory activity. These results may occur because of the presence of other major compounds in the extracts. However, the detection of flavonoids in MPP root and leaf extracts could be partly responsible for anti-hyperuricemic and anti-inflammatory effects of the extracts. Flavonoids are found to have anti-inflammatory, antioxidant and anticancer activities (Lin et al., 2002; Balasundram et al., 2006). Moreover, flavonoids have been reported to be potent plant-based xanthine oxidase inhibitor (Chang et al., 1993; Cos et al., 1998). Previous *in vivo* study reported that kaempferol (100 mg/kg) and quercetin (100 mg/kg) significantly (*p* ≤ 0.05) reduced serum uric acid in potassium oxonate-induced hyperuricemic mice (Zhu et al., 2004; Haidari et al., 2009). Lee and Choi (2010) showed that myricetin (10 μM) significantly reduced IL-1β-induced secretion of IL-6 and MMP-1 in synovial cells SW982. In another study, Huang et al. (2012) reported that quercetin (400 mg/kg) downregulated the levels of IL-1β, TNF-α, COX-2, and PGE_2_ in the serum, liver and joint synovial tissue of rats induced with MSU crystals.

Several natural products have been reported to exhibit the anti-inflammatory effect of MSU-induced inflammation *in vivo*. Most of natural products that were reported to possess anti-inflammatory activity against MSU-induced inflammation contained active components, such as phenolics, flavonoids, terpenoids and steroids. These compounds have been reported to possess anti-inflammatory activity. Huang et al. (2012) reported that quercetin at doses of 200 and 400 mg/kg reduced edema, decreased histological sign of acute inflammation in MSU-induced inflammation in rats. Additionally, quercetin also decreased the recruitment of leucocyte, cytokine and chemokine levels, lipid peroxidation end-product malandolialdehyde and increased anti-oxidant enzyme activity in rats. Thus, the presence of these components in *M. pumilum* could be responsible for the anti-inflammatory activity against MSU-induced inflammation.

Since several flavonoids such as myricetin, quercetin and kaempferol were detected in the extracts, the anti-hyperuricemic and anti-inflammatory activities of the extracts might be contributed by these compounds. However, as mentioned above, most of flavonoids in *M. pumilum* were flavonoids glycosides which reported to possess lower anti-hyperuricemic and anti-inflammatory activities compared to flavonoid aglycones (Teng and Chen, 2018). Hence, it is believed that the presence of other major compounds in the extracts may also contributed either in inhibiting or enhancing the activities. However, additional studies are required to isolate and identify the bioactive components present in MPP roots and leaves.

## Conclusion

In conclusion, this study demonstrated that MPP leaf extract possess high *in vitro* XO inhibitory activity. *In vivo* study on hyperuricemic rats showed that the MPP leaf extract reduces serum uric acid level of hyperuricemic rats and exhibits liver XO inhibitory activity. It is suggested that the *in vivo* anti-hyperuricemic effects of MPP leaves may be partly through liver xanthine oxidase inhibitory activity and we speculated that there are other mechanism underlying the anti-hyperuricemic activity of MPP such as uricosuric activity. Thus, further investigation on this activity would be highly recommended. The *in vitro* anti-inflammatory assays revealed that extract of MPP roots showed the anti-gouty inflammatory activity by inhibiting IL-1α, IL-1β, IL-6, IL-8, TNF-α, and PGE_2_ secretion in MSU-induced PBMCs. The *in vivo* study showed that extract of MPP roots inhibited IL-1α, IL-1β, IL-6, TNF-α, and PGE_2_ secretion. Therefore, MPP root is a promising candidate for developing as treatment for gouty inflammation.

The overall results indicate that MPP has potential as a treatment for gout in term of its anti-hyperuricemic and anti-inflammatory activity, despite it was from different part of plant. The anti-hyperuricemic effect was achieved by XO inhibitory activity or maybe in synergy with other mechanism such as uricosuric activity. Meanwhile, the anti-inflammatory activity was shown by the inhibitory activity of MSU-induced cytokines and PGE_2_ secretion. To the best of our knowledge, this is the first report about dual actions of MPP as anti-hyperuricemic and anti-inflammatory. This research provides a basis knowledge to develop new anti-gout therapy which attenuate both hyperuricemia and inflammation response.

## Data Availability Statement

All datasets generated for this study are included in the article/Supplementary Material.

## Ethics Statement

The studies involving human participants were reviewed and approved by the Human Ethical Committee of Universiti Kebangsaan Malaysia. The patients/participants provided their written informed consent to participate in this study. The animal study was reviewed and approved by the Universiti Kebangsaan Malaysia-Animal Ethics Committee (UKMAEC).

## Author Contributions

ER was the master candidate who conducted the experimental works and drafted the manuscript. JAJ was the project leader responsible for the research design and editing of the manuscript. EK was a project team member who contributed ideas for the research design and reviewed the manuscript. JJ and KH are project team members who contributed ideas for the research design. FB was veterinarian who helped with the *in vivo* assay. AR provided the technical assistance for *in vitro* anti-inflammatory assay.

## Conflict of Interest

The authors declare that the research was conducted in the absence of any commercial or financial relationships that could be construed as a potential conflict of interest.
