# Supplementary material for: Anti-hyperuricemic and Anti-inflammatory Effects of Marantodes pumilum as Potential Treatment for Gout
Source: Front Pharmacol. 2020 Mar 17;11:289. doi: 10.3389/fphar.2020.00289 (PMC7092620; doi:10.3389/fphar.2020.00289)
Supplement: Supplementary file 1 [file Data_Sheet_1.docx]

Supplementary Material

# Supplementary Figure


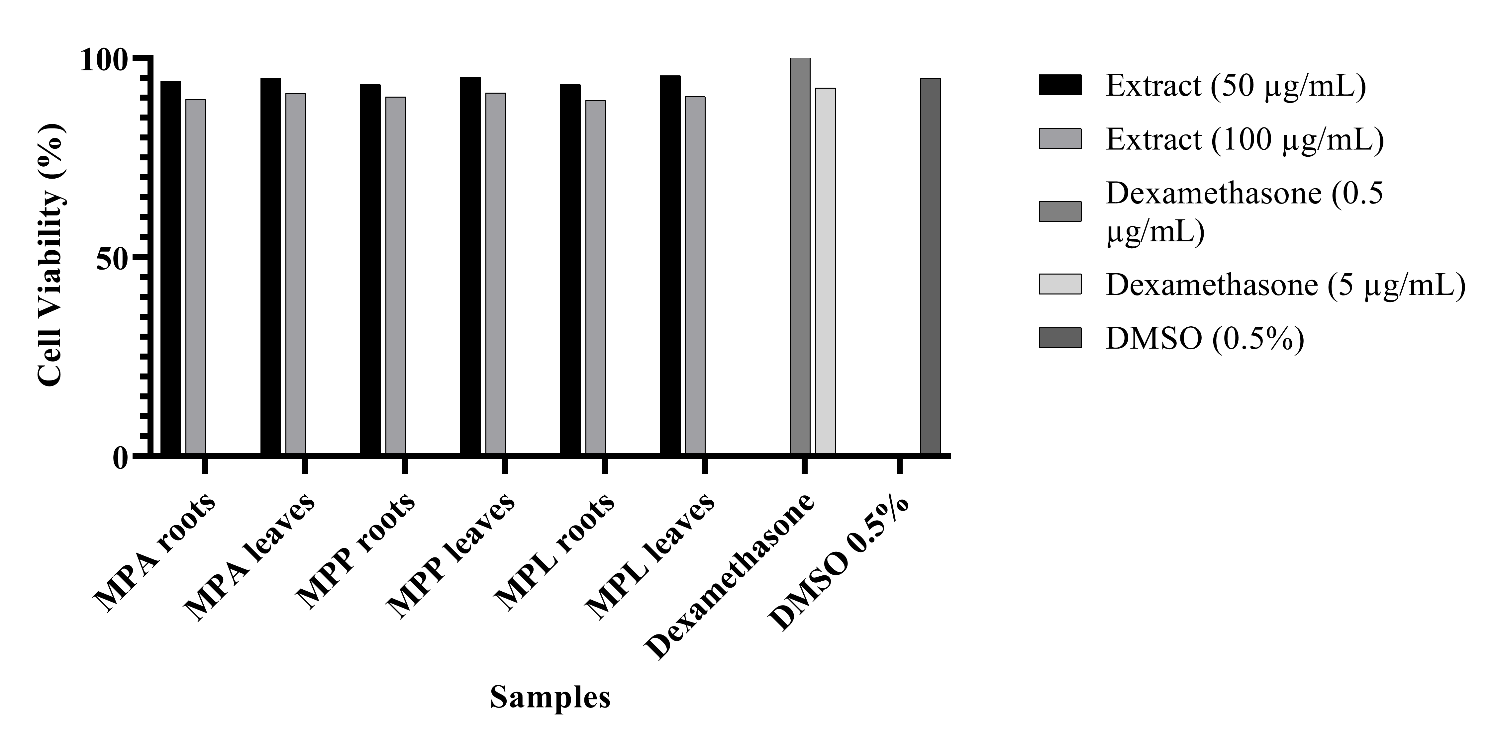


Supplementary Figure 1 Viability of PBMCs after 27 h of exposure to extracts of *Marantodes pumilum* and reference standard. Data are presented as mean±SEM (n=3).

**2. Supplementary Table**

Supplementary Table 1 Full Taxonomic Name of the Plant

| **No** | **Plant common name** | **Plant full scientific name MANDATORY DATABASE (The Plant List)** |
| --- | --- | --- |
| 1 | Kacip Fatimah | *Marantodes pumilum* (Blume) Kuntze |
